# Extreme root resorption associated with induced tooth movement: A protocol for clinical management

**DOI:** 10.1590/2176-9451.19.5.019-026.oin

**Published:** 2014

**Authors:** Alberto Consolaro, Laurindo Zanco Furquim

**Affiliations:** 1 Full professor, School of Dentistry,University of São Paulo/Bauru. Professor, Postgraduate Program, School of Dentistry, University of São Paulo/ Ribeirão Preto; 2 Associate professor, Orthodontics, State University of Maringá (UEM)

**Keywords:** Tooth resorption, External apical resorption, Apical resorption, Orthodontic resorption, Apical rounding, Short roots

## Abstract

Cases in which teeth have only the cervical third remaining from orthodontically induced external root resorption, cast the following doubts: 1) What care should be taken to keep these teeth in mouth with the least risk possible? 2) What care should be taken with regards to reading of imaging exams, particularly in terms of accurately determining cervical root and bone loss? 3) Why is not endodontic treatment recommended in these cases? The present study aims at shedding light on the aforementioned topics so as to induce new insights into the theme.

## Abstract (pt)

Nos casos em que os dentes têm apenas o terço cervical remanescente de uma reabsorção radicular externa induzida ortodonticamente: 1) Quais seriam os cuidados para que permaneçam na boca, com o menor risco possível de perda? 2) Quais seriam os cuidados na interpretação imaginológica desses casos, quanto à determinação precisa da perda radicular e óssea cervical? 3) Por que o tratamento endodôntico não estaria indicado? No presente trabalho, procura-se esclarecer esses pontos e respondendo os questionamentos, para induzir novos insights sobre o assunto.

Tooth resorption is present in 5 to 10% of the general population who has never been subjected to orthodontic treatment. It has been considered the major cause of tooth loss; however, considerable confusion remains with regards to diagnosis of the different types of tooth resorption.

Dental trauma is the major cause of tooth resorption leading to tooth loss. Nevertheless, from an epidemiological standpoint, its most frequent cause is orthodontic treatment which hardly ever induces resorption leading to tooth loss. Thus, the present study aims at discussing the following issues so as to provide guidelines for future clinical protocols:

◆ In which case teeth reabsorbed during orthodontic treatment are considered lost?
◆ What is the limit of resorption-induced root tooth loss required for extraction?

## Orthodontically induced resorption is always inflammatory

Tooth resorption is grouped according to seven different criteria,^1^ each one of which assigns an appropriate and distinct term for a number of categories. In terms of mechanism of occurrence, tooth resorption is mainly classified into two types as follows: 

1 - **Inflammatory resorption:** When, in the process of resorption, periodontal space and ligament remain inflamed and act as the source of mediators for the cells to resorb mineralized root tissue. The major causes of inflammatory resorption are orthodontic movement, periapical periodontitis, trauma and inside bleaching. 

From a cellular standpoint, the cause of periodontal inflammatory process leads to local cementoblasts death, even though periodontal ligament vitality is preserved and so are the epithelial rests of Malassez - the structure responsible for keeping the periodontal space uniform, thereby avoiding alveolodental ankylosis. Orthodontic treatment induces this type of resorption.

2 - **Replacement resorption:** This type of resorption only occurs as a result of alveolodental ankylosis resulting from trauma of erupted teeth or severe atrophy of the periodontal ligament in unerupted teeth. Teeth undergoing alveolodental ankylosis are considered a bone structure. For this reason, they are subjected to constant renewal processes and remodeling, and are replaced by bone. Alveolodental ankylosis only occurs in the event of death of epithelial rests of Malassez, an epithelial structure of the periodontal ligament which is not affected by orthodontic treatment.

## Root is reabsorbed by periodontal ligament!

Except for cases of internal resorption (a specific form of resorption that represents pulp pathosis), teeth are always reabsorbed by the periodontal ligament. Resorptive cells have access to mineralized dental tissues via periodontal ligament which also consists of resorptive cells.

In external tooth resorption, the pulp does not undergo any inflammatory, aging or mineralization processes. Additionally, it does not show any symptoms or potential for necrosis. In other words: Removing the pulp during endodontic treatment does not affect speed and severity of external tooth resorption induced by orthodontic movement. Endodontic post-treatment phase might be a complicating factor due to providing risk of contamination and filling material overflow in cases of orthodontically-induced resorption.

Endodontic treatment interferes in tooth resorption induced by inflammatory processes caused by bacteria in the root canal and/or dentin tubules. In these cases, endodontic treatment does imply in eliminating or controlling the cause of resorption. 

Using calcium hydroxide to endodontically treat teeth with external root resorption induced by chronic periapical periodontitis or pulp necrosis - including by-products adhered to the root structure - implies in making good use of its antimicrobial and anti-inflammatory properties, especially its alkalizing effect. This is due to the bacteria that characterize this microbiota, as they tend to prefer acid environments to proliferate and metabolize. Calcium hydroxide alkalizing effect is temporary and does not interfere in other resorptive activities, except for microorganism-induced inflammation.

## Periodontal support: essentially cervical!

Tooth root apex accounts for 10% of periodontal support. Thus, losing the apical third does not significantly affect periodontal support. This explains why some researches on orthodontic techniques and other variables yield final outcomes with apparently severe apical loss which is not subject to questioning by scientific reviewers. One might assert, from a periodontal support standpoint, that the apex is unimportant.

The middle third of the root accounts for 30% of periodontal support. This means that a tooth with half root preserved remains with 70% of periodontal support. Conversely, the cervical third of the root accounts for 60% of periodontal support due to being of greater proportions, with greater diameter and circumferential perimeter. Nature might have designed it this way to compensate for greater alveolar bone elasticity or deformation capacity in a more cervical level, which provides the tooth-bone complex with greater stability.

In practical terms, this means that a tooth with only the cervical third of the root left might perfectly remain in one's mouth, performing its masticatory, speech and esthetic functions without increased mobility or further gingival alterations. 

## What is the best procedure for teeth with only the cervical third left?

Once again: In practical terms, a tooth with only the cervical third of the root left might perfectly remain in one's mouth, performing its masticatory, speech and esthetic functions without increased mobility or further gingival and color alterations.

From a preventive point of view, the clinician must ask the patient to do the following:

1. Avoid grasping food with your teeth, only, so as to prevent excessive tension.
2. Use individual acrylic plates while sleeping so as to ensure that forces exerted by clenching and/or bruxism during sleep concentrate on some teeth, only.
3. Use a mouthpiece while doing sports, since teeth might undergo avulsion even if exposed to minor trauma.
4. In order to prevent further issues, a retainer should be used so that, in the event of trauma or negligence in day-to-day life, these short-rooted teeth have greater strength.

## Endodontic treatment should not be performed in these cases!

Teeth with severely resorbed roots must never be subjected to endodontic treatment with a view to stopping resorption or rendering the tooth resistant to it: That does not occur!

When faced with emergency situations, many clinicians lose control over logical and biological reasoning, and randomly appeal to all possible alternatives, even improper and ineffective ones. 

Orthodontically induced resorption is controlled if force is removed: After seven days, there will be no more clasts, and after four to five weeks, the entire root surface will be restored with new cementum and periodontal fibers. Root resorption is achieved by periodontal ligament, not by dental pulp. Endodontic treatment is useless in these situations. After six weeks, tooth length stability is restored.

## From a periodontal point of view

Should there be tooth mobility, it does not result from tooth resorption. Tooth mobility might be caused by:

◆ Orthodontic forces, whether active or residual.
◆ Occlusal trauma superimposed over resorptive processes.
◆ Cervical bone loss of orthodontic and iatrogenic nature.
◆ Exuberant cervical bone loss associated with periodontal disease or occlusal trauma.

To correct tooth mobility, its cause must be detected and properly solved. Should the pulp be vital, it must not be subjected to endodontic treatment due to potential mobility; unless pulp and periapical space need to be handled for surgical purposes.

## Imaging aspects of root resorption and cervical bone

Surprisingly, periapical radiograph is more reliable than tomography in cases requiring precise details. 

**Figure 1:**
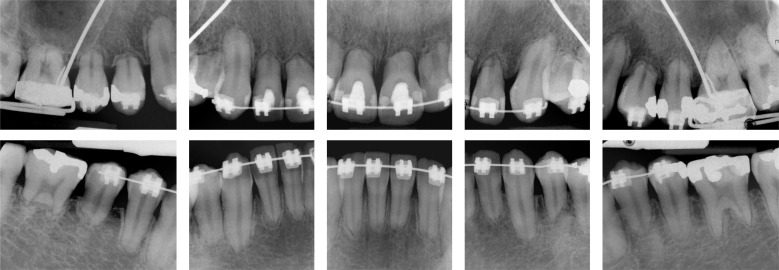
Severe inflammatory root resorption (Malmgren's grade 4) after four years of orthodontic treatment. Most teeth, including first molars, have only the cervical third remaining. Note the detailed root and bone structures involved in the resorption process.

**Figure 2:**
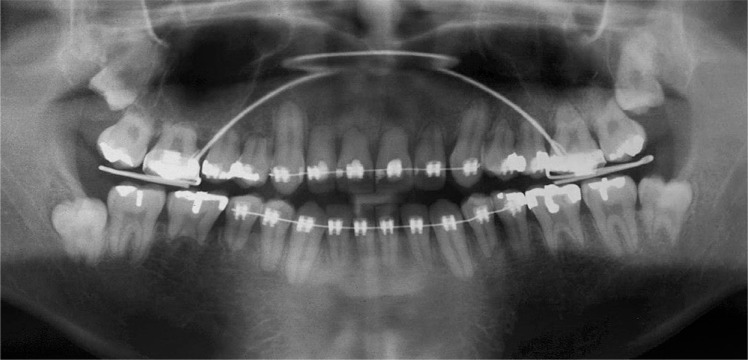
For comparative purposes, note that conventional panoramic radiograph reveals the same severe inflammatory root resorption (Malmgren's grade 4) after four years of orthodontic treatment

**Figure 3:**
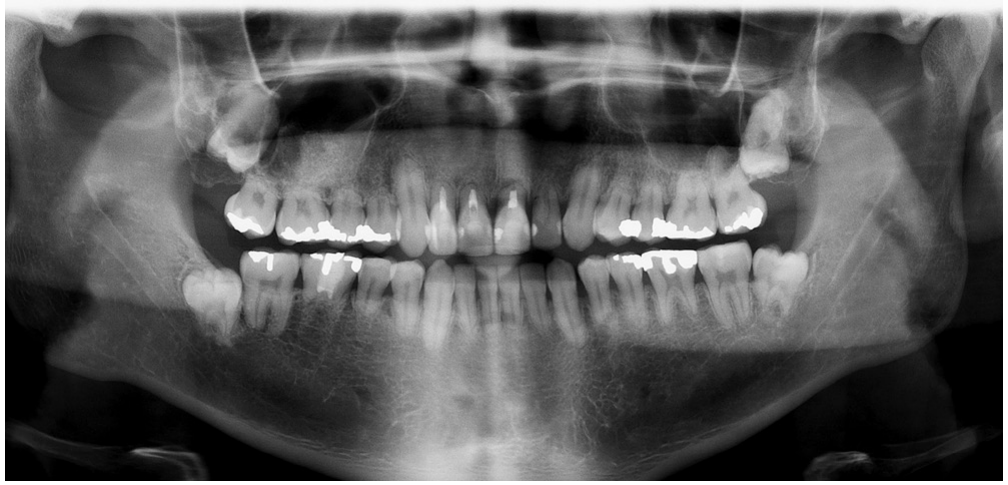
After four years of orthodontic treatment, some severe inflammatory root resorptions (Malmgren's grade 4) were mistakenly treated by means of endodontic therapy as revealed by digital panoramic radiograph.

**Figure 4:**
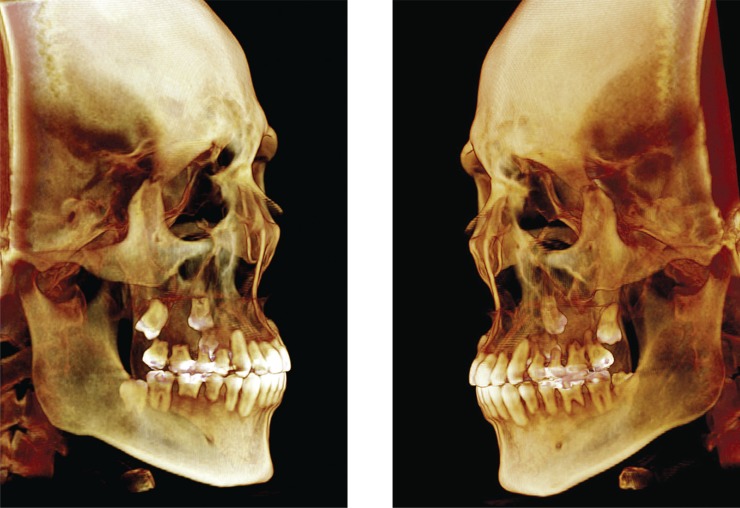
Severe inflammatory root resorption (Malmgren's grade 4) revealed by 3D tomographic scans that allow a contextual and comparative assessment of the process in each tooth and their respective surfaces

**Figure 5:**
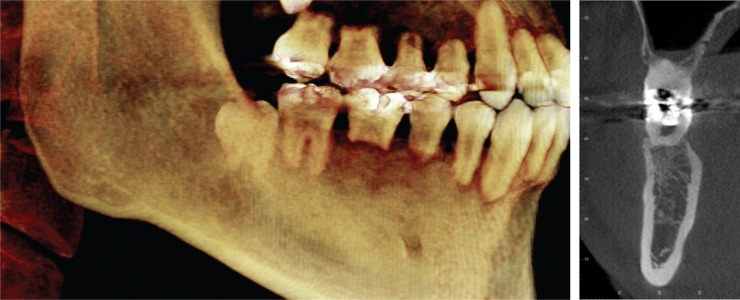
Severe inflammatory root resorption (Malmgren's grade 4) after four years of orthodontic treatment, including premolars and molars. Apparently, teeth have no bone or alveolar cortical bone support; however, periapical radiograph reveals detailed root and bone structures involved in the resorption process. Thus, it is reasonable to assert that there exists periodontal support provided by the cervical third.

**Figure 6:**
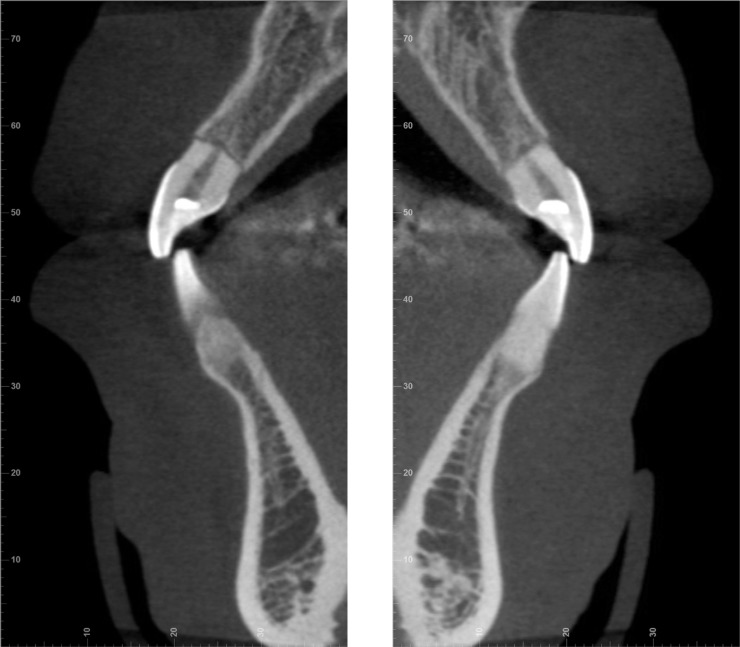
Tomographic slices of severe inflammatory root resorption (Malmgren's grade 4) after four years of orthodontic treatment. Apparently, teeth have no bone or alveolar cortical bone support; however, periapical radiograph reveals detailed root and bone structures involved in the resorption process. Thus, it is reasonable to assert that there exists periodontal support provided by the cervical third.

Thinner cortical bones and delicate trabeculae tend not to appear in tomographic slices or panoramic radiographs. A thorough analysis comparing Figures 1 to 6 illustrates such a fact. 

## Clinical case analysis

**Figure 7:**
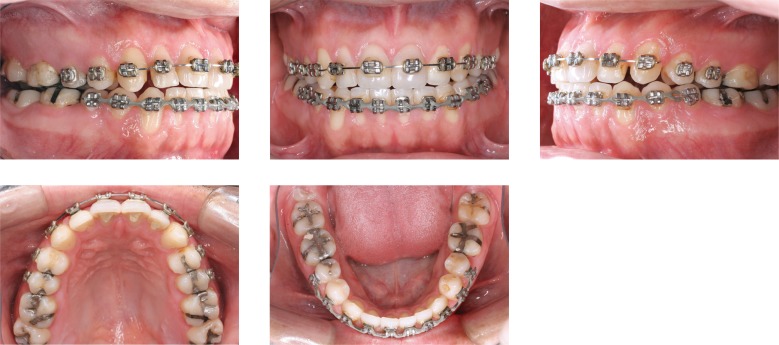
Clinical dental and gingival aspects of patient presenting with severe inflammatory root resorption (Malmgren's grade 4) after four years of orthodontic treatment.

A mongoloid, 25-year-old male patient sought orthodontic treatment after being subjected to a four-year therapy, as revealed by Figures 1 to 7. Although he had most roots with severe resorption (Malmgren's grade 4),^2^ no teeth should have been extracted or submitted to endodontic procedures. Nevertheless, three maxillary incisors underwent endodontic treatment which, in fact, does not affect root prognosis. 

Dental pulp does not influence external resorption. Similarly, intracanal dressing does not interfere in the cause of resorptive processes while active forces remain. Conversely, removing forces does cause resorptive processes to stop in such a way that no more clasts are found on the root surface after a week.

During the first appointment, after four years of previous orthodontic treatment, patient's teeth did not show increased mobility. Occlusion assessment revealed absence of incisal guidance and Class III relationship between canines and molars (Fig 7). As stated by the patient, there was an ongoing attempt to correct Class III by means of elastics: Intermittent forces such as those exerted by intermaxillary elastics might favor root resorption during orthodontic treatment. 

**Figure 8:**
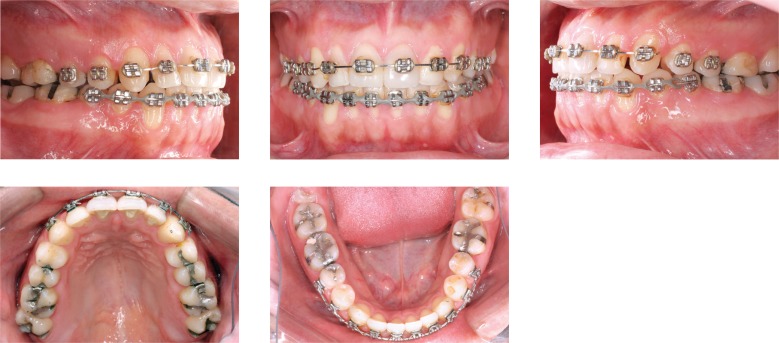
Clinical aspect after occlusal adjustment: incisal guidance and improved canines and molars occlusion.

The orthodontic appliance was not removed and occlusal adjustment was carried out to improve occlusion (Fig 8). After three months of assessment and once new occlusal wear was carried out, the brackets were removed and the patient subjected to quarterly, semestral and annual control. 

**Figure 9:**
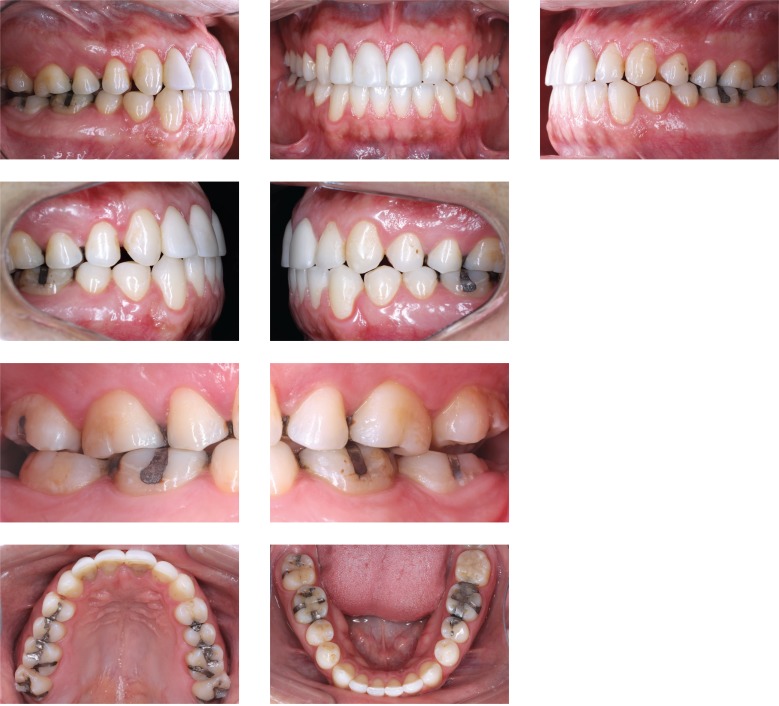
Clinical dental and gingival aspects of patient presenting with severe inflammatory root resorption (Malmgren's grade 4) after applying the protocol suggested with a 6-year control.

Occlusal interference associated with tissue lesions caused by occlusal trauma might lead to increased tooth mobility and ongoing root resorption, even after orthodontic forces are removed.

Considering the severity of root resorption and the conditions of remnant cervical root (responsible for 60% of periodontal support), the following procedures must be followed:

1^st^ Teeth can remain in function and esthetics for an indefinite period of time without endodontic treatment, except for cases in which endodontic therapy is exclusively required. In the case reported herein, endodontic treatment proved unnecessary (Figs 1 and 3).

2^nd^ Occlusion must be thoroughly balanced without further interference. Should there be any type of interference, they must be immediately corrected as in the case reported herein. 

3^rd^ The patient should be advised to use a mouthpiece while practicing sports. In the event of a trauma occurs, clinicians should follow the same procedure employed for teeth without root shortening. 

4^th^ Making patients aware that while eating, they should avoid grasping hard food, such as some fruit or bread, with their teeth, only. 

5^th^ In cases of bruxism, even if mild and occasional, the patient should ideally, routinely and methodically use individual acrylic plates while sleeping.

6^th^ Since roots are too short, tooth movement should be avoided.

7^th^ Should movement be exclusively orthopedic without involving compromised teeth and their anchorage, the periodontal ligament is not affected by inflammation or stress. In other words, orthopedic movement does not induce a new cycle of root resorption.

8^th^ Chronic inflammatory periodontal disease associated with dental plaque must be prevented by properly advising the patient about oral hygiene. Minor cervical bone loss is utterly significant. 

9^th^ Fully or partially unerupted teeth must be extracted, especially if they are too near other teeth which might not only lead to root resorption, but also hinder the case due to orthodontic reasons.

10^th^ Parafunctional habits, such as onychophagia, object grasping with teeth, labial or lingual piercings, must be corrected and avoided.

## How probable is tooth loss? When should a retainer be used?

Should the aforementioned care be taken, the probability of tooth loss is significantly reduced. Likewise, should proper care be taken, the need for a retainer is also reduced. 

Should there be tooth mobility, the clinician must question its causes which might be related to occlusal trauma, chronic inflammatory periodontal disease or cervical bone loss associated with root resorption. In these cases, the cause must be eliminated and potential sequelae corrected. Importantly, tooth mobility might be among such sequelae; however, not due to root resorption in which case the use of a retainer is necessary.

## Where do pulp and periodontal ligament cells of resorbed roots go to?

Pulp and periodontal ligament are special connective tissues that undergo renewal processes during the day, in addition to adapting to the demands of each area. 

Connective tissue cells are stable and, whenever faced with mediators such as normal growth factors, join the cell cycle and are differentiated into similar cells, (fibroblasts, osteoblasts and chondroblasts) via mitosis. Some of those cells are undifferentiated or pre-differentiated, and have great differentiation capacity for other morphologies and functions.

## Final considerations

Teeth with only the cervical third remaining from orthodontically induced external root resorption must remain in one's mouth with function and esthetics preserved. In these cases, endodontic treatment is not recommended for affected teeth because the pulp is not involved in the process and the post-treatment phase of endodontic therapy might be a complicating factor due to risks of accidental contamination or filling material overflow. 

Accurate diagnosis of causes and stages of development, in addition to occlusal trauma control and oral hygiene as well as the use of a mouthpiece to avoid trauma and acrylic plates to correct bruxism are part of the protocol recommended to treat cases of extreme root resorption associated with induced tooth movement. 

Additionally, care should be taken with regards to reading of imaging exams, since tomography does not accurately reveal minor details of thin cortical bone and trabeculae. Periapical radiograph, on the other hand, provides precise details, especially in terms of detecting cervical bone and root loss. 

Should proper care be taken by clinicians and patients, the chances of tooth loss in extreme cases of root resorption associated with induced tooth movement are reduced.
